# Lessons Learned in Digital Health Promotion: The Promise and Challenge of Contextual Behavioral Science Methodology in Valuing Intervention Research

**DOI:** 10.3390/bs15081095

**Published:** 2025-08-12

**Authors:** Jessica M. Criddle, Wesley Malvini, Hayley Jasper, Michael J. Bordieri

**Affiliations:** 1 Department of Psychology, Murray State University, 401C Wells Hall, Murray, KY 42071, USA; jcriddle@positivedevelopment.com; 2 Department of Psychology, University of Louisiana at Lafayette, Lafayette, LA 70503, USA; wesley.maxwell-malvini1@louisiana.edu; 3 Department of Education and Psychology, University of Texas at Tyler, BEP 223, 3900 University Blvd., Tyler, TX 75799, USA; hjasper@patriots.uttyler.edu

**Keywords:** values, mHealth, physical activity, diet quality, sleep, alcohol use, ecological momentary assessment

## Abstract

Using individualized approaches leads to longer-term pro-health behavior change. Both technological delivery methods and values-centered Acceptance and Commitment Therapy (ACT) are useful frameworks for personalized interventions. This investigation sought to explore the effects that valuing had on health using an internet-delivered audio and writing group-level intervention. Specifically, we replicated the use of domain-specific outcomes and idiographic motivational statements sent via text message while additionally employing individualized intervention delivery components, objectives, and statistical methods. While this intervention did not generate significant improvement in health behaviors relative to a control in a sample of 107 college student participants, it has implications for future digital health intervention design and implementation as well as the further development of theoretically consistent valuing research methods.

## 1. Introduction

College students in the United States face significant health risks, including problematic substance use, poor nutrition, low sleep quality, and low rates of exercise (Lawrence, 2017; University of Minnesota, 2015). Given the many behavior patterns in college students that can lead to unwanted outcomes (e.g., American College Health Association, 2021; Becker et al., 2018; Gray & Squeglia, 2017; Lederer & Oswalt, 2017; Reilly & Kelly, 2010; Simmonds et al., 2015; van der Zee et al., 2019), it is essential to develop accessible resources and interventions for health in the college population.

Many prevention and intervention approaches have been developed to address such health risks, with varying effectiveness. Public health approaches often use informational interventions, which include free resources such as health screenings and community health literacy, as low health literacy has been shown to correlate with poorer health outcomes (Fan et al., 2021). Yet, interventions disseminating health information typically do not result in significant behavior change unless given in one-on-one clinical settings (Kahwati et al., 2016; Ross & Melzer, 2016). In fact, recent systematic reviews show that more effective informational interventions are more likely to intervene using multiple simultaneous strategies and individualized intervention aspects such as behavioral counseling, self-reflection on motivation, and self-affirmation (Sheeran et al., 2017; Stormacq et al., 2020). The benefits of an individualized approach are also demonstrated in exercise science research exploring motivation, such that higher levels of behavioral engagement and long-term change are correlated with intrinsic health motivation and personally chosen behavioral intentions (e.g., Kane et al., 2004; Teixeira et al., 2012; Webb & Sheeran, 2006). Such interventions may include motivational interviewing and programs or smartphone applications (apps) with tailored feedback (Lustria et al., 2013; Morton et al., 2014). Recent trends in health research are exploring apps with personalized feedback and practitioner-led personalized medicine to increase preventative health behaviors, which further individualize interventions (Buford et al., 2013; Ghanvatkar et al., 2019).

Studies and meta-analysis also demonstrate significant health behavior change from values-centered studies, indicating the efficacy of personalized interventions focusing on motivation (Epton et al., 2015; Levin et al., 2021). Additionally, meaning in life has been shown to correlate with physical activity, sleep quality, and lower rates of alcohol and drug use (Csabonyi & Phillips, 2017; Hooker & Masters, 2014; Hooker et al., 2020; Kim et al., 2015). Despite these relationships, there has been limited attention given to values- based health interventions for the college population. One useful framework for development of interventions to increase healthy behaviors and reduce unhealthy behaviors is Acceptance and Commitment Therapy (ACT).

### 1.1. ACT and Valuing

ACT is a third wave behavioral therapy, the central aim of which is not to reduce human suffering, as suffering is assumed to be a typical aspect of the human experience (Fung, 2014). Alternatively, ACT-based approaches seek to change a person’s relationship with suffering and self (Wilson & Murrell, 2004) and to imbue life with meaning through mindful actions in line with one’s values (Harris, 2019; Michelson et al., 2011). The overarching model for this change has been coined *psychological flexibility* (PF; Hayes et al., 2006), which entails acceptance and awareness of one’s own thoughts, feelings, and emotions, and working toward living in line with values even in the presence of aversive contingencies (Hayes, 2004; Hayes et al., 2012). The six core processes of PF are *contacting the present moment*, *defusion*, *acceptance*, *self-as-context*, *values*, and *committed action* (Harris, 2019). Values provide meaning in life and direction in the use of the other processes, while committed action refers to patterns of successful behavior associated with personal values (Hayes et al., 2012).

In ACT, values are conceptualized as, “freely chosen, verbally constructed consequences of ongoing, dynamic, evolving patterns of activity, which establish predominant reinforcement for that activity that are intrinsic in engagement in the valued behavioral pattern itself (Wilson & DuFrene, 2009, p. 66).” Unlike goals, which can be completed, valuing is an ongoing process (Chase et al., 2013; Hayes et al., 2012). Values are constructed on a moment-to-moment basis and change over time, as engagement with valued action will influence one’s future construction of valuing in the same area. These valued actions are reinforcing in and of themselves, with behaviors under appetitive instead of aversive control (Smout et al., 2014). Engagement in valued behavior also broadens one’s behavioral repertoire, leading to expanding patterns of behavior which allow for increasing engagement in even more valued actions (Louisiana Contextual Science Research Group, 2022).

### 1.2. Valuing and Rule-Governed Behavior

From a behavior analytic approach, instances of *valuing* are *rule-governed behavior* (RGB), which is controlled by verbally mediated consequences, as opposed to behavior shaped by environmental contingencies with which we come into direct contact (Törneke et al., 2008). One functional class of RGB is *pliance*, defined as behavior controlled by socially mediated consequences, wherein another person both establishes the rule and delivers the consequence, directly connecting the rule and the behavior that will result in those consequences (Kissi et al., 2017). Pliance may also be directed by self-established rules with socially mediated consequences such as social approval (Törneke et al., 2008). For example, a person may go to the gym because they receive social acceptance and praise by doing so. Inherent in this conceptualization is behavior under the control of socially mediated consequences, which is not congruent with the CBS conceptualization of valuing, wherein behavior is under the control of the correspondence between a verbal rule (e.g., a self-generated value statement) and a pattern of action (Plumb et al., 2009).

### 1.3. Valuing in Research

Several recent studies collecting longitudinal ecological momentary assessment (EMA) data on valuing each demonstrates the dynamic nature of valuing and its role in both day-to-day and longer-term health outcomes. Grégoire et al. (2021) found wellbeing and distress were more accurately predicted by day-to-day valued actions than averaged valued living, and that higher daily variability predicted increasing distress over time. Finkelstein-Fox et al. (2019) and Berghoff et al. (2018) found that different psychological flexibility processes discriminatively predicted daily valued living, with the former showing day-to-day within-person variance in valued action as an indicator of daily psychological health. Together, these studies show that small, consistent behaviors in line with one’s values can increase wellbeing and health most effectively over time. However, valued actions also have immediate benefits and other aspects of psychological flexibility that influence our ability to engage in these actions in the moment. Many other ACT interventions also show that valuing may be the mechanism through which multiple predictors result in favorable outcomes (Michelson et al., 2011; Vowles & McCracken, 2008; Wallin et al., 2018; Wersebe et al., 2017).

Two recent studies show promise in more personalized research regarding health values-consistent behavior. Stapleton et al. (2020) used health specific values to predict health outcomes in college students rather than using general valued living. General values-consistent living and psychological flexibility were measured using the Valued Living Questionnaire and CompACT, respectively. Outcomes included survey measures for physical activity (International Physical Activity Questionnaire; Craig et al., 2003), dietary quality (Diet Quality Tool; O’Reilly & McCann, 2012), sleep quality (Pittsburgh Sleep Quality Index; Buysse et al., 1989), alcohol consumption (Quick Drinking Screen; Sobell et al., 1996), and cigarette consumption (adapted from the National Health Interview Survey; Centers for Disease Control and Prevention, 2016), all which are areas that are identified by the Centers for Disease Control and Prevention (2019) as key health components. Health values-consistent living predicted higher sleep quality but no other outcomes. General values-consistent living predicted more physical activity and better sleep quality but not diet quality or cigarette and alcohol consumption. The authors concluded that abstract values were not enough to predict behavioral outcomes, and that value measurement and behavioral outcomes should match in specificity.

Another recent study (Jackson et al., 2016) sought to ensure a functional value–behavior relationship using a specific, pre-set target behavior participants’ chose themselves (a university cycling class) while allowing them to state their own personal motivation for said behavior. Participants rated a list of 24 common health and aesthetic related reasons why one might engage in physical activity and then constructed their own personal statement expressing what motivates them to exercise. Their own statement and the six highest rated statements from each category were used to create an *Implicit Relational Assessment Procedure* (IRAP; Barnes-Holmes et al., 2006) unique to each participant. The IRAP is a computerized procedure based on Relational Frame Theory’s (RFT) *derived relational responding* principle which allows for identification of implicit relational preferences, and the results were used to validate the selection of values statements used in the experimental condition. Participants attended 10 cycling exercise classes and were given either a statement they had indicated was in line with their motivation for health (e.g., “*to fit into my skinny jeans*”) or a statement containing an instructional message (e.g., “*push through your heels*”) with an assignment utilizing an alternating treatment design. They found that when participants had a statement they rated as motivating presented to them their heart rate was significantly higher compared to presentation of an instructional statement and to baseline. These findings reflect the concept of valuing, as verbal establishing operations influenced behavior by connecting it to consequences. Furthermore, motivational statements differed by individual, and the reinforcing consequences linked to these statements were often temporally distal while cycling (e.g., “*to fit in my skinny jeans*”).

### 1.4. Implications for Novel Research Methods

Jackson et al. (2016) and Stapleton et al. (2020)’s methods have implications for valuing studies, as survey measures assessing broad, abstract values do not steadily predict engagement in target behaviors (Barrett et al., 2019; Dahl, 2015). One reason for this disconnect may be that researcher-generated target behaviors may not hold the same evocative effect and connection to that value for each or every participant (Hayes, 2004). Additionally, one feature of valuing as conceptualized in ACT is that the reinforcer for valued behaviors is self-selected for its inherently reinforcing nature to an individual, rather than socially mediated through compliance with researcher expectations (Bond, 2004; Dahl, 2015). Researcher-decided target behaviors and valued domains may induce participants to perform due to pliance (Kissi et al., 2017; Zettle & Hayes, 1982), and researchers should seek to mitigate some of this effect with design elements.

An individualized approach such as in the Jackson et al. (2016) study can navigate some of these difficulties by having participants create a motivational statement that is more likely to be intrinsically reinforcing and encouraging them to connect target behaviors to their valuing in a functional manner. Most research on valuing to date has utilized traditional group-level design, such as self-report questionnaires and an intervention applied to a group, despite the idiographic nature of valuing. Stapleton et al. (2020) and Jackson et al. (2016)’s studies establish techniques to make group-level design more congruent with the nature of valuing by including individualized intervention elements and connecting valuing to one specific domain (i.e., health).

There are multiple ways to individualize participants’ values and target behaviors in group designs. One of these is the implementation of a front-end intervention to guide participants’ engagement with values as freely chosen and not as socially desirable (e.g., fusion with socially desirable values). A second method is narrowing down the valued domains to one domain associated with target behaviors (e.g., health) and keeping data from participants who rate this as highly valued (Stapleton et al., 2020). A third method is to broadly assess multiple target behaviors within a valued domain to explore participants’ unique pattern of behavior change. Fourth, and most importantly, allowing participants to construct their own personal valuing statement around this domain instead of choosing from a researcher-generated list will assist in encouraging a more direct relationship between valuing and target behaviors. These methods allow this analogue study for short-term behavior change to examine the mechanism of action involved in such change and the utility of valuing interventions to potentially develop long-term interventions to disseminate to the general college population.

### 1.5. Current Study

This current study explored the influence of a previously validated valuing intervention on health behaviors in a college population. This was conducted through replication and extension of studies exploring health valuing (Jackson et al., 2016; Stapleton et al., 2020). Specifically, this study replicated the multiple domain-specific outcomes demonstrated by Stapleton et al. (2020) and Jackson et al. (2016)’s procedure for generating idiographic motivational statements. In addition, this study extended the use of valuing interventions (Harris, 2008) to health-specific domains. Synthesis of these methods in this analogue study has the potential to encourage increased health behaviors in college students, including physical activity, dietary quality, sleep quality, and alcohol consumption.

The current study had two main hypotheses. First, we hypothesized that participant-selected outcomes of most personal importance would predict higher pre–post change in the related outcome for those in the intervention condition. The intervention condition would show greater standardized improvements in the selected health outcome of the most personal importance relative to pre–post changes in the control condition. Second, we hypothesized that changes in values awareness and engagement would mediate the relationship between intervention condition and the standardized change score in selected health outcome of the most personal importance, with participants in the intervention condition experiencing improvements in their chosen domain via the indirect effect of the valuing process. This analysis was primarily used to assess valuing as the mechanism of change involved in behavior change following the valuing intervention. It additionally provided information on engagement with the intervention. Because ACT interventions elicit change through learning by direct experiences (such as the valuing intervention) and not psychoeducation, changes in valuing itself are more congruent with the ACT model than using attention checks to assess engagement. Exploratory analyses were also conducted to examine differences in health behavior change across health domains at the group level.

## 2. Materials and Methods

### 2.1. Participants

The sample consisted of 107 students enrolled at a university in the midwestern United States. Inclusion criteria for survey participation required participants be age 18 or older and have English language proficiency.

### 2.2. Procedure

All procedures were approved by the university’s Institutional Review Board (Protocol 22-030) prior to data collection and funding was obtained through a university grant via the Murray State University Office of Research and Creative Activity (ORCA Grant #83 & #101). Additionally, this study was preregistered on Open Science Framework (Center for Open Science; https://doi.org/10.17605/OSF.IO/V3QXD) There were two recruitment strategies used: the first was recruitment through undergraduate psychology classes participating in a digital research participant pool across two semesters; the second recruited students from campus through emails to official campus organizations. The samples were pooled due to a similarity in demographics. Participants recruited through the research pool received course credit as a participation incentive. All participants who completed the post-intervention measures were eligible for entry into a drawing for one of five $10 gift cards to an online retailer.

After providing informed consent to participate, participants followed a link leading them to eight psychometric battery measures, which took approximately 30 min to complete. Participants then continued to the intervention portion of the study. Random assignment to an intervention condition (values-based or informational) was automated by the online open-source Lime survey platform v3.26 (LimeSurvey GmbH, n.d.). After intervention and control procedures, all participants selected the health domain of most importance to their own health from a list of the domains assessed in this study.

Participants were then invited to sign up for ecological momentary interventions (EMI) over text. During the 10-day intervention window, participants assigned to the intervention condition received a text daily at 10:00 a.m. that contained their personally crafted motivational statement. Participants assigned to the informational control condition received texts each morning with information regarding a domain of health being assessed. At the end of the 10-day intervention window participants in both conditions were presented with the same battery of questionnaires given at baseline, excluding the demographic survey. They then completed a program evaluation to assess the perceived benefits and usability of the intervention.

### 2.3. Intervention and Comparator

#### 2.3.1. Valuing Intervention Condition

Participants assigned to the values condition listened to a 4 min audio recording during the initial study timepoint guiding them in the process of valuing as conceptualized in ACT, differentiating values from goals, and giving an example of valuing. Examples from the recording include “*values are directions we keep moving in, whereas goals are what we want to achieve along the way*” and “*they are leading principles that can guide us and motivate us as we move through life*.”

The audio recording was adapted from two widely used clinical tools created by ACT practitioners (Harris, 2008; Wilson & Murrell, 2004): the Values Worksheet and the Sweet Spot exercise. As per the Values Worksheet, a focus of the recording was valuing as a dynamic, evolving process (e.g., “*Values are directions we keep moving in, whereas goals are what we want to achieve along the way.*”). It stressed valuing as freely chosen and differing from person to person (e.g., “*not everyone has the same values, and this is not a test to see whether you have the “correct” values.*”). The script was further tailored to those who value health by giving an example of possible health values and by including questions from the Values Worksheet and the Sweet Spot exercise that engage participants in actively thinking about what might personally motivate them to engage in healthy behaviors (e.g., “*How do you want to look after your health, with regard to sleep, diet, exercise, smoking, alcohol,* etc.*? Why is this important?*”). This recording was followed by a list of values to aid in their consideration; all specifically related to health (e.g., fitness, empowerment, accomplishment; Stapleton et al., 2020).

Next, participants were given the textual prompt, “What are your values related to maintaining your physical well-being? How do you want to look after your health, regarding sleep, diet, exercise, smoking, alcohol, etc.? Please list your own personal reasons for choosing healthy behaviors. Be specific.”. A free response text box was provided. This item is consistent with the methods used in Jackson et al. (2016) and Harris (2008) and supplied an idiographic (i.e., specific to that individual) statement on values surrounding physical health for each participant.

#### 2.3.2. Informational Control Condition

To give the control condition an equivalent time spent in the study, participants randomized to the control group listened to an approximately 4 min audio recording. The content included statements from credible health organizations detailing recommendations for ideal dietary quality, sleep quality, physical activity, cigarette consumption, and healthy alcohol consumption levels. The health psychoeducation procedure is in line with Jackson et al. (2016)’s informational exercise statements in their control condition and provides data on how individualized motivational statements affect health behaviors above and beyond health literacy. Time equivalence was ensured through word count compared to the intervention condition (i.e., approximately 500 words).

#### 2.3.3. Daily Interventions

Starting approximately one week after completion of baseline measures and the intervention or control recordings, values intervention participants received a morning text containing the motivational statement they constructed. Participants began the texting phase of the intervention between one and six days following the initial study timepoint. Informational control condition participants received a morning text containing instructional, actionable statements from respected organizations on how to improve that domain of health (e.g., “*Look for opportunities to reduce sedentary time and to increase active time. For example, instead of watching TV, take a walk after dinner.—CDC*.”). These informational texts were designed to be consistent with the instructional statements seen in Jackson et al. (2016). All texts were sent through CallFire (CallFire Inc., 2024; 1410 2nd St Suite 200, Santa Monica, CA, USA), a secure, professional texting service.

### 2.4. Measures

Demographics Questionnaire. A short questionnaire gathered self-report data on demographics including age, ethnicity, gender and sexual identity, and college class.

Valued Living Questionnaire (VLQ; Wilson et al., 2010). The VLQ assesses participants’ values and how they are living in accordance with them across 10 common life domains (e.g., family, career). Participants were asked to rate the importance of each domain and how consistently they have acted in accordance with their values in the past week on a 10-point scale, with higher scores indicating a greater importance in an area and greater belief one is living in line with one’s values (*α* = 0.86 in the current sample). Psychometric evaluation has found acceptable internal consistency (*α* = 0.67–0.79) test–retest reliability (*r* = 0.74–0.76), and convergent and discriminant validity (Cotter, 2011).

Valuing Questionnaire (VQ; Smout et al., 2014). The VQ is a two-factor, self-reporting measure that assesses progress and obstruction to valued living. *Progress* is defined as engagement with valued living, awareness of values, and perseverance. *Obstruction* is defined as lack of engagement with valued behaviors due to avoidance or inattention to values. The VQ shows good convergent and discriminatory validity and good internal reliability (*α* = 0.79–0.81; Smout et al., 2014). Higher scores in the two separate progress and obstruction subscales indicate higher engagement with, or barriers to, valued living, respectively (*α* = 0.80; *α* = 0.81 in the current sample).

Nicotine Consumption. Nicotine consumption was assessed using one item taken from the *National Health Interview Survey* (Centers for Disease Control and Prevention, 2016). Participants reported frequency of current cigarette and e-cigarette smoking behaviors on a scale of 0—*Not at all* to 2—*Every day*. No Cronbach’s alpha was calculated for cigarette consumption because it contains a single item.

Diet Quality Tool (DQT; O’Reilly & McCann, 2012). The DQT is a 13-item self-reporting measure that assesses the intake of important dietary nutrients by combining information on the number of servings of four food groups (e.g., fruit) and the quality of seven food groups (e.g., grain). Criterion and construct validity were acceptable for use, with overall DQT scores correlating with fat, fiber, and omega-3 scores (*r* = −0.50, *r* = 0.55, and *r* = 0.32, respectively). A higher score indicates eating habits that are more in line with nutritional guidelines (*α* = 0.77 in the current sample). Wording has been replaced with American English where necessary to ensure comprehension by the sample (e.g., “*biscuits*” changed to “*cookies*”).

Pittsburgh Sleep Quality Index (PSQI; Buysse et al., 1989). Sleep quality was evaluated with the PSQI. Participants rated seven components of sleep quality and disturbances (e.g., duration, daytime dysfunction) in 19 items. The component subscales are summed for a total score, with lower scores indicating higher sleep quality (*α* = 0.76 in the current sample). The PSQI demonstrates good internal consistency reliability (*α* = 0.70–0.80) and good convergent and discriminant validity (Buysse et al., 1989; Carpenter & Andrykowski, 1998).

Quick Drinking Screen (QDS; Sobell et al., 1996). The QDS assesses unhealthy alcohol consumption in five areas: average number of days drinking per week, average drinks per week, average number of drinks when drinking, frequency of binge drinking occurrences, and the highest number of drinks consumed on one occasion. The QDS was originally developed to estimate these factors over the last 90 days. While no test–retest reliability is available, interclass correlations with a previously validated standard drinking measure were all significant (*p* < 0.001) across all domains. The QDS was also found to be more reliable than another commonly used brief measure, the three-item Alcohol Use Disorders Identification Test—Concise (see Letourneau et al., 2017; Sobell et al., 1996). No Cronbach’s alpha was calculated due to only being a single item.

International Physical Activity Questionnaire—Short Version (IPAQ; Craig et al., 2003). The IPAQ was designed to evaluate physical activity. Participants rate nine items that collect information on intensity of physical activities (slow, moderate, vigorous, and walking) and how often they were sedentary in the past seven days. It is a psychometrically sound measure, with good inter-method concurrent validity with the IPAQ long form (*r* = average of 0.80) and acceptable reliability (*r* = 0.35–0.88, with ¾ of items above 0.65). Number of days spent in a type of activity multiplied by the number of minutes is calculated for each intensity level and the scores for each intensity level are summed. Higher scores indicate higher levels of physical activity (*α* = 0.69 in the current sample).

Assessment of Health Behavior Importance. Health behavior importance was assessed using one item that required participants to choose the domain of health most personally important to them. Options included alcohol use, nicotine use, diet quality, sleep quality, and physical activity. Due to a programming error, this measure was not administered to some participants during the initial stages of data collection.

Program Evaluation. A series of questions asked participants to rate aspects of the interventions to inform the possible development of tools to increase health in college populations. Quantitative items include the helpfulness of the intervention regarding behavior engagement and increased awareness of personal health valuing, both on a five-point scale. Qualitative items included the question “*What did you like or not like about the daily text messages?*” and an open text box for any other feedback.

### 2.5. Power Analysis

A priori power analyses were conducted using G*Power 3.1 (Faul et al., 2009) with α = 0.05 and 1 − β = 0.80. Research Question 1 (ANCOVAs for health domains): 112 participants were required to detect a medium effect and the obtained sample of 107 was slightly underpowered. Hypothesis 1 (ANCOVA): 112 participants were required and the H1 sample (*N* = 74) was underpowered. Hypothesis 2 (mediation model approximated as multiple regression with three predictors): 119 participants were required and the H2 sample (*N* = 74) was underpowered.

### 2.6. Statistical Methods

All cleaning, coding, and analyses of data was conducted using IBM SPSS Statistics (Version 25). For the sake of brevity, a more detailed description of methods used for data preparation, other analytic procedures, baseline analyses, and non-key findings are available for access elsewhere. The original study may be reviewed at no charge at https://digitalcommons.murraystate.edu/etd/257/ (accessed on 16 April 2025). Additionally, the dataset and additional materials can be accessed on the Open Science Framework’s data repository at https://osf.io/hzk9n/ (accessed on 16 April 2025) (Supplementary Materials).

## 3. Results

### 3.1. Study Flow and Baseline Descriptives

Of the 252 participants who completed the baseline survey, 118 did not complete the second timepoint and were removed. The overall attrition rate from baseline to post-intervention surveys was 46.8%, attrition in the valuing condition was 43.6%, and attrition in the informational condition was 49.6%. A Fisher’s exact test revealed no significant effect of condition on attrition between participants allocated and retained for analysis, *p* = 0.818. All cases with more than 10% of missing responses were removed (*n* = 13) with one instance of a duplicate response and one participant in the valuing condition was discontinued due to a value statement suggesting potentially restrictive eating behaviors. Participants with a score of five or less on the VLQ were also removed prior to analysis (*n* = 11). While not pre-registered, this criterion was established prior to data collection to ensure participants valued health a priori and not due to implied researcher requirements (i.e., pliance).

**Figure 1:**
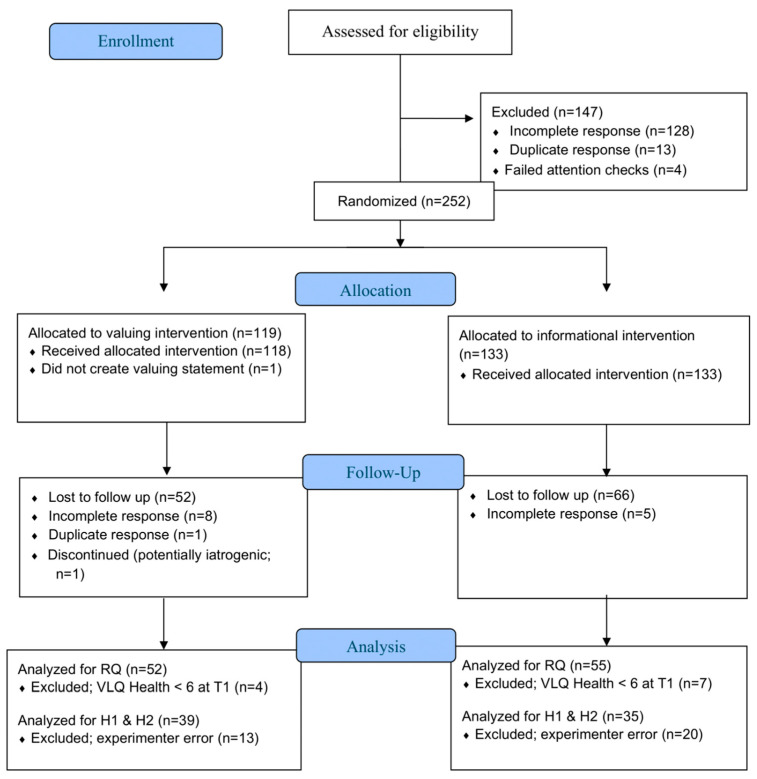
CONSORT Flow Diagram.

The final study sample size was 107, with 52 valuing and 55 information condition participants analyzed. Unfortunately, due to a technical error, the assessment of health behavior importance measure was not properly administered to 33 participants (Valuing *n* = 13; Informational *n* = 20), leaving data from only 74 participants available for the analysis of Hypothesis 1 and 2. See the CONSORT diagram (Figure 1) for details on participant enrollment, assignment to condition, attrition at each stage of the study, and analyses.

Most participants (*N* = 107) identified as female (*n* = 71, 66.4%), heterosexual (*n* = 84, 78.5%), and White (*n* = 91, 85%). The average age of the sample was 20.3 years of age (*SD* = 6.00). Participants in the valuing condition (*n* = 52) and informational condition (*n* = 55) showed similar demographic characteristics (see Table 1). Descriptive statistics and correlations were calculated for all study variables at baseline (Table 2).

### 3.2. Change in Valued Health Behavior

The intervention condition was hypothesized to show greater standardized improvements in the selected health outcome of the most personal importance relative to pre–post changes in the control condition. This hypothesis was tested using an analysis of covariance (ANCOVA). With the intervention condition as the independent variable. The dependent variable was a standardized (expressed as *z*-scores) pre–post intervention change score in the participant’s selected health domain with change scores calculated such that positive scores reflected a positive change in health. The standardized baseline score for the relevant health domain was entered for each participant as a covariate. There was no significant effect of intervention condition on health behavior change in participant-selected domain after controlling for baseline scores in participant-selected domain, *F*(1, 73) = 0.40, *p* = 0.531, *ηp*^2^ = 0.006.

### 3.3. Mediation by Values Awareness and Engagement

Changes in values awareness and engagement were hypothesized to mediate the relation between condition and the change score in selected health outcomes of most personal importance. Intervention conditions were entered as the predictor and the standardized, pre–post intervention change score in participants’ selected health domain and were entered as the dependent variable for both groups. Pre–post-intervention change score for values progress from the VQ was entered as the mediator. The standardized baseline score for the selected health domain was entered as a covariate.

**Figure 2:**
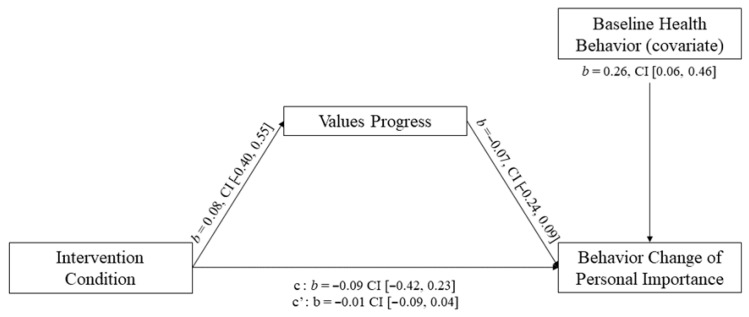
Mediation Results for Hypothesis 2.

Results showed that intervention conditions were not associated with a change in values progress (*b* = 0.08, *SE* = 0.24, *t* = 0.32, *p* = 0.749; Figure 2). Values progress was not associated with a change in health behavior in the domain of most personal importance (*b* = −0.07, *SE* = 0.08, *t* = −0.93, *p* = 0.351). A bootstrap estimation approach was used to compute standardized indirect effects for each of 10,000 bootstrapped samples, with 95% accelerated confidence intervals used to determine significant paths. The indirect effect was not significant, (95% CI [−0.09, 0.04]), indicating a lack of mediation by a change in values progress.

### 3.4. Overall Health Behavior Improvement

The impact of the intervention conditions across all health domains was tested using a series of six ANCOVAs to determine if the values intervention led to greater general improvements in health. The independent variable was the intervention, and the dependent variables were each health domain at post-intervention (T2). For all analyses, the covariates were baseline scores for the corresponding health domain. The effect of the intervention condition on health behavior change while controlling for baseline score was not significant for all dependent variables. See Table 3 for ANCOVA results. See Table 4 for means and standard deviations by condition.

### 3.5. Program Evaluation

Participants rated the helpfulness of the interventions and the subjective increase in values awareness on scales of one to five. Helpfulness ratings had a mean score of 3.66 (*SD* = 0.92). The valuing (*M* = 3.70, *SD* = 0.90) and informational (*M* = 3.60, *SD* = 1.00) conditions had similar ratings, *t*(105) = 0.54, *p* = 0.589, *d* = 0.11. Overall evaluation of an increase in values awareness had a mean of 3.33 (*SD* = 0.97). The valuing participants rated a greater increase in values awareness (*M* = 3.60, *SD* = 1.00) relative to informational participants (*M* = 3.20, *SD* = 0.90), *t*(105) = 2.17, *p* = 0.032, *d* = 0.42. Two items asked participants, “*What did you like and not like about the daily texts?*” and “*Please give any other feedback you chose or type n/a.*” Response statements were categorized by theme as per the qualitative content analysis method (Bengtsson, 2016). See Table 5 for frequencies in all categories.

## 4. Discussion

An ACT-congruent valuing intervention to increase health behaviors in college students was compared to a traditional informational intervention. Previous research points to both values-based and personalized interventions as providing positive effects on behavior change (Epton et al., 2015; Gregg et al., 2014; Jackson et al., 2016; Levin et al., 2021; Lillis et al., 2021). Such effects were not observed in the current study, with participants given the valuing intervention not showing greater changes in positive health behaviors in domains of the most personal importance relative to participants assigned to the informational intervention. Nor did changes in values progress mediate the relation between intervention conditions and changes in health domains of most personal importance. Additionally, there were no significant differences in overall health behavior change between interventions.

Both the quantitative and qualitative program evaluation data indicate that there are favorable elements in the study design, showing moderate social validity and acceptability. Results indicated that participants found the interventions motivating, good reminders of their goals and values, personalized, to contain valuable information, and generally positive. In addition, participants in the valuing condition reported significantly higher post-intervention ratings of values awareness than those in the informational condition, indicating that the valuing intervention effectively enhanced participants’ awareness of their health values.

This preliminary finding is tempered by other program evaluation outcomes. While multiple participants (*n* = 6) in the valuing intervention indicated their intervention was motivating, more of those assigned to the informational condition (*n* = 10) expressed being motivated. This is problematic given that values are conceptualized as motivational augments in ACT (Kissi et al., 2017; Smout et al., 2014; Zettle & Hayes, 1982). Many participants created statements containing behavioral goals rather than valuing statements, such as, “*I would like to get back to going to bed at 10 and getting up at 8 every day to get back on a sleep schedule.*” A contrasting example of values-oriented content includes, “*I want to be able to challenge myself on a regular basis.*” Future interventions might be designed to delineate the difference between values and goals in a more in-depth manner, possibly with multiple exemplars rather than one (e.g., Krafft et al., 2017; Rahal & Gon, 2020). Further, the current exercise could be expanded so that participants have a longer period in which to interact with the content and receive benefits (Li et al., 2022).

Another limitation of the current intervention design was the absence of personalized feedback regarding the values statements generated in the valuing condition. This intervention was based on clinical tools typically used in an in-person setting, wherein the client has access to feedback and ongoing construction of values through discussion (Lillis et al., 2021). Inclusion of telehealth or other one-on-one initial communication options may help people who require assistance in detecting adaptive reinforcing opportunities, discriminating behavior needed to contact reinforcers, tacting appropriate contingencies, or shifting perspective (Hayes & Strosahl, 2013). Artificial Intelligence (AI) is one possible route to real-time, personalized feedback without a clinical provider (e.g., Heinz et al., 2025). For example, younger populations will use AI conversational agents frequently while using mHealth apps, resulting in high engagement (Vertsberger et al., 2022). These have recently been shown to be effective with an ACT intervention (e.g., Naor et al., 2022). However, there are still questions on the veracity and helpfulness of AI in mHealth to ensure quality care (Deniz-Garcia et al., 2023).

One strength of the current design is that participants interacted with the researcher only in digital formats, chose their health domain of interest, and were only included in analyses if they rated health importance as 6 out of 10 or greater. These design features reduced the potential impact of pliance as an alternative source of behavioral control. However, the evocative effect of values statements in this study may not have been as salient as other uncontrolled sources of behavioral control. Valuing interventions need to target significant behaviors with strong reinforcing functions (Fryling, 2012; Whelan & Barnes-Holmes, 2004). It is possible that the physically healthy function targeted in the current study may not have been as salient as other reinforcers, including course credit for research participation. Participants received course credit before completing the second timepoint, which may have promoted attrition. Further, the reinforcing value of course credit may have competed with the motivating augmental instead of having an additive effect. This is not necessarily detrimental, as academic success is a useful and workable goal for students. However, it suggests that future research in this domain may consider only recruiting college students who express an interest in improving health for its own sake.

The obtained attrition rate of 46.8% is on the higher end of average rates in the reported literature on technologically delivered health trials, which typically fall between 26% and 49%, and up to 60% or more (e.g., Howarth et al., 2018; Meyerowitz-Katz et al., 2020; Prior et al., 2023). However, health behavior change has higher rates of adherence than other mobile interventions and is not often maintained in naturalistic settings (Amagai et al., 2022). While the broader literature helps place the observed attrition rate in context, the loss of nearly half of the sample to follow-up remains a significant concern. Unfortunately, the generally favorable program evaluation data come only from participants who completed the study, so it is possible that the high attrition rate may also reflect feasibility and acceptability concerns related to the digital intervention. Future research in this domain should explore user feasibility more closely by capturing feasibility ratings throughout data collection and not only at the last timepoint.

Related to the attrition concern, the underpowered analyses are also a significant limitation of the current study. The research questions exploring overall improvements across health domains were close to being adequately powered (107 participants obtained with 112 required). Thus, we interpret these null findings as evidence that the digital valuing intervention did not produce statistically significant improvements in health behaviors relative to the informational control in this sample. In contrast, the analysis of participant’s health domain of greatest importance (H1) and mechanisms of change (H2) were clearly underpowered (74 participants obtained with 112–119 required), and we interpret these null findings as inconclusive. Taken together, these results suggest that the current digital intervention did not improve health behaviors broadly at the group level, and that future research with larger, adequately powered samples is necessary to clarify effects and mechanisms at the level of individually selected health domains (Bujang et al., 2017; Shieh, 2019).

### 4.1. Broader Methodological Lessons Learned

Future research should strengthen congruence between study design and a contextual behavioral science (CBS) framework. This study included one personalized intervention (i.e., valuing statement) and one personalized design (i.e., the participant-selected behavior domain of most personal importance). Utilizing single-subject designs (SSD), more longitudinal data collection, network analysis, more precise measurement of processes of change variables in studies (Gates & Hellberg, 2022) could allow for deeper CBS analyses.

#### 4.1.1. Intervention and Study Delivery

Daily EMIs through text could be explored as an adjunct to campus therapy and medical centers—a method proven useful in clinical disorders (Heron & Smyth, 2010; Newman et al., 2015). Many doctor’s offices and hospitals are now offering Focused Acceptance Commitment Therapy (FACT; Glover et al., 2016; Kanzler et al., 2022), in which medical providers are trained to provide one session ACT interventions focused on changing health behavior through the valuing process, and such services could address these difficulties. These approaches could additionally reduce the burden on these facilities and offer daily support to clients, as well as providing support following the termination of treatment.

Additionally, future studies on health behavior change may consider the limitations of self-report, such as the subjective nature of responses, recall bias, and social desirability (Gmel & Daeppen, 2007; Sallis & Saelens, 2000). There are several methods that can help buffer these concerns, including using EMA (K. E. Smith & Juarascio, 2019) and measuring a permanent product of health behavior through technologies such as fitness tracker watches and mobile phone applications (Chow & Yang, 2020).

#### 4.1.2. Intervention Dosing

While there is evidence that 10-day EMI dosage used in the current study can lead to positive outcomes (Jackson et al., 2016; Jeffers et al., 2019), increasing the duration of EMI delivery may increase intervention effectiveness. Another way to increase EMI potency would be to build on ACT-based self-help programs and mobile phone applications with established evidence of efficacy (Bricker et al., 2014; Potts et al., 2020). Interactive content, such as micro-engagement with technologically delivered content, has also been found to increase the effectiveness of interventions (Voorheis et al., 2022).

#### 4.1.3. Analytic Approaches

Starting valuing intervention development at the single-subject level would allow for comparison of response patterns across multiple participants where individual context and characteristics can inform decisions on how, why, and for whom interventions are successful (Hofmann et al., 2020). The additional inclusion of processes of change variables at the individual or group level can allow researchers to determine what biopsychosocial processes maintain or change health-related behaviors over the course of an intervention (Hayes et al., 2021). Interventions can then be modified to enhance or omit elements that target specific mechanisms that are or are not driving behavior change. Group level studies could also provide opportunities for statistical methods such as dynamic network analysis and Group Iterative Multiple Model Estimation, which can be used to combine idionomic and nomothetic approaches (Hofmann et al., 2020; Jordan et al., 2020; Sanford et al., 2022).

#### 4.1.4. Program Evaluation Implications

Participants responded positively to the medium of text, indicating that future research should continue to explore this delivery method. Texting is also accessible, cost-effective, and easy to implement as compared to in-person services (Gaziano et al., 2015; D. M. Smith et al., 2020). Mobile phone prompts for self-guided interventions or fitness and diet tracking applications could also provide a means to increase engagement with content. A bottom-up method of co-creating intervention content and delivery could help ensure engagement and satisfaction of participants (Arevian et al., 2018; Cyril et al., 2015; Walser et al., 2024; Wright et al., 2016).

## 5. Conclusions

There is a dearth of research exploring CBS-informed valuing interventions for health behavior change, especially in college populations. This study compared a valuing intervention to a traditional informational intervention to address this need. The non-significant results highlight the importance of incorporating student feedback and co-development of the design of such interventions. Further, these results point to a need for pulling from design theory with traditional development approaches to better tailor interventions for college populations. Technologically delivered health behavior change interventions can be explored as an adjunct to therapy to enhance the effectiveness of both therapeutic treatments and EMIs. Furthermore, study methods should include research design measures, data collection strategies, and analytic methods that reflect the idiographic nature of the valuing process in affecting personal change.

## Figures and Tables

**Table 1:** Demographic variables by condition at baseline for final study sample (*N* = 107).

Characteristic	Full Sample (*n* = 107)	Valuing (*n* = 52)	Informational (*n* = 55)
Age *M* (*SD*)	20.30 (12.26)	19.96 (5.42)	19.78 (4.2)
**Gender Identity *n* (%)**			
Cisgender Woman	71 (66.4)	36 (65.6)	35 (67.3)
Cisgender Man	31 (29.0)	15 (27.3)	16 (30.8)
Trans Woman	1 (0.9)	0 (0.0)	1 (1.9)
Trans Man	1 (0.9)	1 (1.8)	0 (0.0)
Nonbinary	2 (1.9)	2 (3.6)	0 (0.0)
**Sexual Identity *n* (%)**	1 (0.9)	1 (1.8)	0 (0.0)
Heterosexual	84 (78.5)	38 (69.1)	46 (88.5)
Bisexual	11 (10.3)	7 (12.7)	4 (7.7)
Lesbian	2 (1.9)	1 (1.8)	1 (1.9)
Gay	4 (3.7)	3 (5.5)	1 (1.9)
Pansexual	3 (2.8)	3 (5.5)	0 (0.0)
No Answer	3 (2.8)	3 (5.5)	0 (0.0)
**Race/Ethnic Identity *n* (%)**			
White/European American	91 (85.0)	48 (87.3)	43 (82.7)
Black/African American	9 (8.4)	4 (7.3)	5 (9.6)
Latin/a/o/x	9 (8.4)	3 (5.4)	6 (10.8)
Asian/Asian American	4 (3.7)	1 (1.8)	3 (5.8)
Indigenous Tribes/First Peoples/Native American	1 (0.9)	1 (1.8)	0 (0.0)
More than one race/mixed race	1 (0.9)	1 (1.8)	0 (0.0)
Not Listed	1 (0.9)	1 (1.8)	0 (0.0)

**Table 2:** Descriptives and correlation coefficients between study variables at baseline.

Variable	1	2	3	4	5	6
1. Values Progress (VQ-P)	-	0.17	0.06	−0.02	0.09	−0.30 **
2. Physical Activity (IPAQ)		-	−0.08	0.20	−0.04	−0.11
3. Nicotine Consumption			-	−0.13	0.26 **	0.11
4. Diet Quality (DQT)				-	−0.05	0.06
5. Alcohol Use (QDS)					-	0.06
6. Sleep Quality (PSQI)						-
*M*	20.10	657.30	0.40	40.00	3.40	6.70
*SD*	4.90	1232.20	0.70	18.10	7.30	3.10

Note. ** *p* < 0.01, *N* = 107, VQ-P = Valuing Questionnaire-Progress subscale; IPAQ = International Physical Activity Questionnaire; DQT = Diet Quality Tool; QDS = Quick Drinking Screen; PSQI = Pittsburgh Sleep Quality Index.

**Table 3:** Results of the between-group analysis of change in all health domains with baseline value of constructs entered as a covariate.

Health Domain	*F*	*p*	*η_p_* ^2^
**Physical Activity (IPAQ)**			
Baseline	260.15	<0.002 *	0.714
Condition	3.68	0.058	0.034
**Nicotine Consumption**			
Baseline	6.66	0.011 *	0.060
Condition	0.01	0.936	0.000
**Diet Quality (DQT)**			
Baseline	4.79	0.031 *	0.044
Condition	0.92	0.340	0.009
**Alcohol Use (QDS)**			
Baseline	98.25	<0.002 *	0.486
Condition	0.11	0.746	0.001
**Sleep Quality (PSQI)**			
Baseline	67.39	<0.002 *	0.393
Condition	0.49	0.484	0.005

Note. * *p* < 0.05, *N* = 107, IPAQ = International Physical Activity Questionnaire; DQT = Diet Quality Tool; QDS = Quick Drinking Screen; PSQI = Pittsburgh Sleep Quality Index.

**Table 4:** Pre- and post-test descriptive statistics for overall health behavior change by condition.

Health Domain	Valuing (*n* = 52)		Informational (*n* = 55)		
	***M***	***SD***	***M***	***SD***	***d***
**Physical Activity (IPAQ)**					
Baseline	672.40	967.70	643.10	1447.70	0.02
Post-test	626.00	843.80	420.60	432.00	0.31
**Nicotine Consumption**					
Baseline	0.40	0.80	0.40	0.70	0.00
Post-test	0.40	0.70	0.40	0.80	0.00
**Diet Quality (DQT)**					
Baseline	38.60	17.80	41.30	18.40	−0.15
Post-test	40.00	19.10	40.60	18.30	−0.03
**Alcohol Use (QDS)**					
Baseline	3.40	6.30	3.40	8.10	0.00
Post-test	3.20	6.20	2.80	5.20	0.07
**Sleep Quality (PSQI)**					
Baseline	6.20	3.20	7.30	3.40	−0.33
Post-test	4.50	2.30	5.30	2.30	−0.35

**Table 5:** Program evaluation dimension frequencies.

Content	Total	Valuing	Informational
Good reminder	19	15	4
Motivating	16	6	10
Uncategorized	13	5	8
Good information	10	-	10
Good, general	12	6	6
Not applicable to me	8	-	8
Helped with my goals	8	8	-
Felt personalized	10	9	1
Bad, general	6	2	4
Thought provoking	5	4	1
Timing of texts	6	2	4
Ambivalent	6	5	1
Helped valued behavior	5	5	-
Actionable	2	-	2
Unhealthy reminder	2	-	2
Future suggestions	2	-	2

## Data Availability

De-identified data presented in the study are openly available in Open Science Framework at https://osf.io/hzk9n/files/osfstorage (accessed on 16 April 2025).

## Supplementary Materials

Supporting information, including intervention scripts and informational texts, can be downloaded at: https://osf.io/hzk9n/ (accessed on 16 April 2025).
